# Substrate Protein Enrichment of Black Soldier Fly (*Hermetia illucens*) Larvae for Diets of Olive Flounder (*Paralichthys olivaceus*)

**DOI:** 10.1155/anu/4780779

**Published:** 2026-05-22

**Authors:** Seong-Mok Jeong, Byung Hwa Min, Jinho Bae, Mingi Kim, Sang Woo Hur, Kihwan Park, Kang Woong Kim

**Affiliations:** ^1^ Aquafeed Research Center, National Institute of Fisheries Science, Pohang, 37517, Republic of Korea, nifs.go.kr; ^2^ Aquaculture Industry Research Division, South Sea Fisheries Research Institute, National Institute of Fisheries Science, Yeosu, 59780, Republic of Korea, nifs.go.kr; ^3^ Entomo Inc., Cheongju, 28304, Republic of Korea; ^4^ Subtropical Fisheries Research Institute, National Institute of Fisheries Science, Jeju, 63068, Republic of Korea, nifs.go.kr

**Keywords:** body nutrient composition, fatty acid profile, food waste, substrate, sustainable aquaculture

## Abstract

Continued growth of the aquaculture industry requires the identification of novel, cost‐effective protein sources. In this study, we evaluated the potential of black soldier fly (*Hermetia illucens*) larvae (BSFL) reared on protein‐enriched food waste (FW) as an alternative to conventional feed ingredients. In a 12‐days rearing trial, 8‐day‐old BSFL were randomly assigned to three dietary groups: FW alone (CBSFL), FW supplemented with soybean meal (SBM) (SBSFL), or FW supplemented with fish meal (FM) (FBSFL). The larvae were subsequently incorporated into diets for olive flounder (*Paralichthys olivaceus*) in an 8‐week feeding trial to assess dietary efficacy. Larval growth performance was highest in the CBSFL group (*p*  < 0.05). However, crude protein content was significantly higher in SBSFL and FBSFL than in CBSFL, confirming that the rearing substrate directly influences larval nutritional composition. In the 8‐week feeding trial, inclusion of BSFL derived from CBSFL and SBSFL substrates in olive flounder diets did not significantly affect growth performance compared with the control. However, it significantly increased lauric acid and total saturated fatty acid (SFA) content and decreased docosahexaenoic acid (DHA) content. These results reveal that BSFL can serve as an effective protein substitute in olive flounder diets, although a trade‐off exists between productivity and nutritional enrichment. Future studies should focus on optimising substrates and processing technologies to maintain larval productivity while enhancing the nutritional quality (especially the fatty acid profile) of farmed fish.

## 1. Introduction

As the global population has surged in the 21^st^ century, so has the demand for high‐quality animal protein. For seafood, a key protein source, an additional 37 million tons is estimated to be required by 2030 to maintain the current consumption levels [1, 2]. However, traditional capture fishery production has reached its limit and is now in a state of stagnation, making aquaculture the primary means to meet future demands [2–5].

The continued success of aquaculture hinges on a stable feed supply, which accounts for the highest portion of the production costs. Because of their low carbohydrate use, marine fish rely on protein as their primary energy source. Consequently, high‐protein feed supply is an essential prerequisite for growth [6–8]. Fish meal (FM) has traditionally met this protein demand in the aquaculture industry owing to its excellent amino acid profile and high digestibility [9]. However, the dependence of FM on wild‐caught fisheries induces highly volatile supply and price and raises sustainability concerns [10, 11]. To address these challenges and achieve sustainable aquaculture, developing alternatives to FM is essential.

Ongoing research has explored various alternative protein sources, including plants [12] and microalgae [13]. Insects, particularly the black soldier fly (BSF; *Hermetia illucens*), have emerged as promising alternatives that satisfy both sustainability requirements and nutritional value. BSF larvae (BSFL) are highly regarded for their sustainability because of their ability to convert organic waste (such as food scraps) into nutrient‐rich biomass. Furthermore, this species exhibits nutritional potential with high protein and lipid content [14–18]. Typically, BSFL are reared on low‐value organic by‐products to ensure economic viability and productivity, and previous studies have examined the efficacy of soy and fish processing wastes as substrates [19]. However, observations that productivity decreases with increasing inclusion of raw sardine in the substrate despite the concurrent increase in n‐3 highly unsaturated fatty acid (HUFA) content highlight the critical need to determine optimal substrate conditions [20]. Although feeding commercial‐grade FM or soybean meal (SBM) to BSFL is economically impractical for mass production, particularly because of the objective of replacing these ingredients, utilising them in an experimental setting provides a necessary nutritional baseline. Furthermore, should these high‐quality inputs significantly enhance productivity, their potential application should be considered. To date, comparative data on how these standard high‐quality protein sources directly affect larval growth and body composition relative to other substrates remain scarce; therefore, establishing optimal substrate conditions is imperative.

Accordingly, in this study, we aimed to comprehensively evaluate the potential of BSFL reared under various substrate conditions. Specifically, this study investigated the determination of optimal rearing substrates to characterise how they influence the nutritional profile of the larvae. Subsequently, the efficacy of the produced BSFL was assessed as a protein source in the diet of olive flounder (*Paralichthys olivaceus*), because replacing FM for this major East Asian species is critical for the stability and economic viability of the aquaculture industry [21]. Ultimately, these findings inform the development of BSFL‐based products suitable for aquaculture feeds in general, extending beyond their specific application to olive flounder.

## 2. Materials and Methods

### 2.1. BSFL Production

#### 2.1.1. Experimental Substrates and Design

The control substrate used in this study was food waste (FW) sourced from a commercial processing facility in Cheonan, the Republic of Korea. FM and SBM were purchased from commercial suppliers to be used as additives. Table 1 lists the proximate composition of each raw ingredient. The control group (CON) consisted solely of unadulterated FW. The two experimental substrates, one with SBM (SBSFL) and another with FM (FBSFL), were formulated on a wet weight basis to assess the impact of nutrient fortification. These substrates were composed of 70% FW, 15% of the respective additive, and 15% tap water. The final moisture content of all substrates was verified using an MS‐70 thermo‐hydraulic analyser (A&D, Tokyo, Japan), which confirmed that the moisture levels were 82% for the CON substrate and 74% for both the SBM and FM substrates.

**Table 1:** Nutrient composition of the ingredients in experimental substrates.

Components	Experimental substrate		
	FW	SBM	FM
Proximate composition (as basis)			
Moisture	85.3	9.1	6.5
Crude protein	4.5	45.6	68.0
Crude lipids	3.7	1.9	10.6
Ash	1.6	5.9	13.3
Essential amino acids (% of the substrate, dry matter)			
Histidine	2.11	2.49	2.57
Lysine	7.48	6.03	8.35
Phenylalanine	3.37	4.93	4.15
Methionine	1.85	0.75	2.76
Isoleucine	4.36	4.54	4.30
Leucine	7.62	7.60	7.71
Threonine	3.96	3.93	4.43
Valine	5.98	4.82	5.28
Arginine	4.12	6.87	6.17
Non‐essential amino acids (% of the substrate, dry matter)			
Cystine	2.61	0.93	0.76
Glycine	5.85	4.23	6.67
Alanine	6.92	4.38	6.60
Aspartic	9.43	11.18	9.74
Serine	4.28	5.00	4.18
Glutamic	18.6	18.5	13.9
Proline	N.D.	4.37	4.19
Tyrosine	1.66	3.18	3.26
Ammonia	9.81	6.27	4.94
Major fatty acid composition (% of the total fatty acids)			
C14:0	1.35	N.D.	5.95
C15:0	5.30	13.73	3.14
C16:0	17.76	14.14	25.25
C16:1	1.89	N.D.	6.11
C18:0	7.43	4.29	4.36
C18:1n‐9	25.51	15.21	17.18
C18:2n‐6	21.13	45.60	5.31
C18:3n‐3	2.82	7.02	3.51
C20:5n‐3	0.90	N.D.	11.31
C22:6n‐3	1.59	N.D.	8.04
SFA	33.7	32.2	40.3
MUFA	27.4	15.2	25.3
PUFA	27.5	52.6	29.6

Abbreviations: FM, fish meal; FW, food waste; MUFA, monounsaturated fatty acids; PUFA, polyunsaturated fatty acids; SBM, soybean meal; SFA, saturated fatty acids.

#### 2.1.2. Rearing Conditions and Larval Sampling

BSFL, 8 days post‐hatching (mean initial weight: ~0.02 g; mean initial length: ~4 mm), were used for the feeding trial. The experiment was conducted in triplicate, with each experimental group being housed in rectangular plastic tanks (550 mm × 350 mm × 140 mm). Each tank was stocked with ~4000 larvae. The larvae were reared for 12 days in the dark. Throughout this period, the internal temperature was maintained at 30.7 ± 0.5°C (mean ± standard deviation [SD]), and the relative humidity was kept at ~44.8% ± 5.2% (mean ± SD). A total of 9 kg of substrate was provided throughout the 8‐day rearing period. The larvae were fed daily, with the amount of substrate adjusted to their developmental stage. The initial feeding rate was 400 g/d, which was incrementally increased throughout the experimental period to ensure sufficient nutrition for the growing larvae and prevent feed depletion. This feeding phase was followed by a 4‐day fasting period to ensure gut evacuation.

#### 2.1.3. Larval Sampling

At the conclusion of the trial, larvae from each tank were manually separated from the residual substrate, thoroughly washed with tap water, and surface‐dried. The total weight of larvae per tank was measured and recorded as the total larval biomass. For the morphometric analysis, 20 larvae were randomly sampled from each tank (*n* = 60 per experimental group), and their total length and body height were measured using digital Vernier callipers. Following these measurements, the remaining larvae from each replicate were dried in a microwave oven (18 kW) at 80°C for ~11 min, or until the final moisture content was reduced to below 6%. The dried larvae were then ground into a homogeneous powder using a laboratory blender. The powdered samples were stored at −24°C until subsequent proximate composition, fatty acid profile and amino acid profile analyses.

### 2.2. Fish Feeding

#### 2.2.1. Experimental Diets

A total of five experimental diets were formulated to be isonitrogenous (53%) and isolipidic (12%). Their detailed formulations and nutritional compositions (i.e., proximate analysis, fatty acids and amino acids) are listed in Table 2. The CON diet was formulated using FM and SBM as the primary protein sources, wheat flour as the carbohydrate source and soybean oil as the lipid source. To create the four experimental diets, the FM of the CON formula was replaced with two different types of BSFL meal at two inclusion levels: CBSFL (reared solely on FW) and SBSFL (reared on a substrate mixture of 70% FW, 15% SBM, and 15% tap water), 7% and 14%. The proportions of wheat gluten, wheat flour, and soybean oil were adjusted accordingly to maintain the nutritional balance across all diets, resulting in a total of five dietary groups for the feeding trial (that is CON, CBSFL7, CBSFL14, SBSFL7, and SBSFL14). The experimental diets were formulated to meet the n‐3 HUFA requirement reported by Kim and Lee [22].

**Table 2:** Ingredient and nutrient composition of the experimental diets.

Components	Experimental diet				
	CON	CBSFL7	CBSFL14	SBSFL7	SBSFL14
Ingredient composition (%)					
Fish meal^a^	60.0	53.0	46.0	53.0	46.0
Soybean meal^b^	10.0	10.0	10.0	10.0	10.0
CBSFL	—	7.0	14.0	—	—
SBSFL	—	—	—	7.0	14.0
Wheat gluten^c^	—	3.4	6.8	2.8	5.6
Wheat flour^d^	22.5	20.9	19.2	21.3	20.4
Soybean oil^e^	3.5	1.7	—	1.9	—
Vitamin premix^f^	1.5	1.5	1.5	1.5	1.5
Vitamin C	0.5	0.5	0.5	0.5	0.5
Choline	0.5	0.5	0.5	0.5	0.5
Mineral premix^g^	1.5	1.5	1.5	1.5	1.5
Proximate composition (% dry matter basis)					
Dry matter	95.0	93.6	94.4	93.2	92.3
Crude protein	53.0	53.2	53.4	53.4	53.6
Crude lipids	11.4	11.4	11.6	11.5	10.9
Ash	12.4	12.0	11.7	11.9	11.6
Crude fibre	0.45	1.07	1.06	0.99	1.43
Calories (cal/g)	4788	4742	4825	4708	4695
Essential amino acids (% of the diet; dry matter basis)					
Histidine	1.19	1.07	1.17	1.21	1.21
Lysine	3.65	3.11	3.12	3.55	3.50
Phenylalanine	1.93	1.93	1.99	2.06	2.57
Methionine	1.14	0.98	1.04	1.28	1.12
Isoleucine	2.08	1.95	1.93	1.99	2.12
Leucine	3.71	3.78	3.55	3.68	3.56
Threonine	2.05	1.88	1.80	2.00	2.02
Valine	2.89	2.20	2.39	2.44	2.47
Arginine	3.00	2.57	2.69	2.89	2.81
Major fatty acid composition (% of the total fatty acids)					
C12:0	N.D.	10.02	20.05	9.47	19.31
C14:0	3.89	5.18	6.16	4.99	5.99
C16:0	2.99	2.41	2.36	2.07	2.04
C16:1	21.1	20.6	20.1	20.6	19.2
C18:0	3.86	3.88	3.75	3.77	3.72
C18:1n‐9	19.9	18.4	17.1	18.5	16.9
C18:2n‐6	26.2	20.1	13.9	20.9	14.7
C18:3n‐3	4.52	3.54	2.55	3.68	2.82
C20:5n‐3	6.86	6.73	5.79	6.61	6.38
C22:6n‐3	5.35	4.30	3.66	4.40	3.84
SFA	33.4	42.7	53.3	42.1	51.6
MUFA	23.7	22.3	20.8	22.3	20.6
PUFA	42.9	34.6	25.9	35.6	27.7

Abbreviations: MUFA, monounsaturated fatty acids; PUFA, polyunsaturated fatty acids; SFA, saturated fatty acids.

^a^Brown fish meal (Blumar, Santiago, Chile).

^b^Soybean meal (The Feed, Seoul, Republic of Korea).

^c^Wheat gluten (The Feed, Seoul, Republic of Korea).

^d^Wheat flour, Baeksul, CJ Foods, Seoul, Republic of Korea.

^e^Soybean oil, Baeksul, CJ Foods, Seoul, Republic of Korea.

^f^Vitamin mix (Aquabest‐V, FEEDBEST CO., Ltd. Cheonan, Republic of Korea).

^g^Mineral mix (Aquabest‐M, FEEDBEST CO., Ltd., Cheonan, Republic of Korea).

#### 2.2.2. Experimental Fish and Feeding Conditions

Juvenile olive flounder were sourced from the Aquafeed Research Center (Pohang, Republic of Korea). Prior to the experiment, the fish were acclimated to the experimental facilities for 2 weeks, during which they were fed a commercial diet (56% crude protein, 8% crude lipid; Suhyup Feed Co. Ltd., Uiryeong‐gun, Gyeongsangnam‐do, Republic of Korea). Following this acclimation period, 375 fish with an initial mean body weight of 22.62 ± 0.03 g (mean ± standard error [SE]) were randomly distributed into 15 circular 600 L tanks at a density of 25 fish per tank. Throughout the 8‐week feeding trial, fish were hand‐fed twice daily (at 09:00 and 16:00) to apparent satiation. A simulated 14:10 h light and dark (14L:10D) natural photoperiod was maintained. Water temperature and dissolved oxygen were monitored daily using a YSI multiparameter meter (YSI Pro DSS, YSI Inc., USA). The average water temperature was maintained at 20.9 ± 4.8°C (mean ± SD), and dissolved oxygen was maintained at 8.6 ± 2.1 mg/L (mean ± SD) through continuous aeration in each tank.

#### 2.2.3. Fish Sampling

Upon completion of the 8‐week feeding trial, all fish were fasted for 24 h and subsequently anaesthetised in a 100 ppm solution of 2‐phenoxyethanol. Survival rates were determined by counting the final number of fish per tank, and the total tank biomass was weighed to assess growth performance. For biometric analysis, five fish were randomly selected from each tank, and their total length and liver and intestinal weights were recorded. The parameters for growth, feed utilisation, and various biological indices were then calculated according to the following equations. Weight gain (%) = (final body weight – initial body weight) × 100/initial body weight, specific growth rate (%/d) = ([ln final weight – ln initial weight] × 100/d reared), feed conversion ratio = feed intake/wet weight gain, daily feed intake (%) = (feed intake × 100)/([initial body weight + final body weight + dead weight] × d/2), protein efficiency ratio = wet weight gain/protein intake, condition factor (g/cm^3^) = total body weight of fish (g) × 100/total length of fish (cm)^3^, viscerosomatic index = viscera weight of fish × 100/total body weight of fish, and hepatosomatic index = liver weight of fish × 100/total body weight of fish. Finally, dorsal muscle samples were collected from these same fish and stored at −25°C for subsequent analysis.

### 2.3. Chemical and Statistical Analyses

#### 2.3.1. Proximate Composition Analysis

The proximate composition of the experimental diets and dorsal muscle was analysed according to the standard methods of the Association of Official Analytical Chemists [23]. Crude protein (N × 6.25) was measured using an automatic Kjeldahl nitrogen determinator (FOSS 8400, FOSS, Hillerød, Denmark), and crude lipid was analysed using a Soxtec System 2043 (FOSS). The moisture content was determined after drying the samples in a convection oven at 135°C for 2 h, and the ash content was quantified by incinerating the samples in a muffle furnace at 600°C for 6 h.

#### 2.3.2. Bound Amino Acid and Fatty Acid Composition Analysis

For the bound amino acid composition analysis, 20 mL of 6 N hydrochloric acid was added to the sample, which was then vacuum‐sealed and hydrolysed in a dry oven at 110°C for 24 h. The hydrolysate was filtered through a 5A filter paper, and the filtrate was concentrated under reduced pressure at 55°C to completely remove the hydrochloric acid and water. The concentrate was dissolved in sodium citrate buffer (pH 2.2), adjusted to a final volume of 25 mL in a volumetric flask, then filtered through a 0.45 μm membrane filter. The sample was subsequently analysed using an automatic amino acid analyser (Biochrom 30+; Biochrom Ltd., Cambridge, UK).

Total lipids were extracted from BSFL, diet, and dorsal muscle samples using a chloroform/methanol mixture (2:1, v/v) according to the method of Folch et al. [24]. Fatty acid methyl esters were prepared from the lipid extracts via methanolysis and subsequently analysed using a gas chromatograph (Trace 1310, Thermo Scientific, Waltham, MA, USA) equipped with a flame ionisation detector and an SP‐2560 capillary column (100 m × 0.25 mm i.d., 0.2 μm film thickness; Supelco, Bellefonte, PA, USA).

#### 2.3.3. Statistical Analysis

Statistical analyses were performed using SPSS Version 23 (SPSS Inc., Chicago, IL, USA). The assumptions of normality and homogeneity of variances were then verified using appropriate statistical tests (Levene’s tests). Based on the results of these tests, either a parametric or non‐parametric approach was used. For data that met the assumptions, a one‐way analysis of variance was performed, followed by Tukey’s honestly significant difference (HSD) post hoc test to identify specific differences among the groups. For the data that did not meet the assumptions, a Kruskal–Wallis test was used instead. For all analyses, statistical significance was set at *p*  < 0.05. The results are reported as the mean ± SE.

## 3. Results

### 3.1. BSFL Growth Performance

Table 3 lists the growth performance of BSFL reared for 13 days on different substrates. The total larval biomass was significantly decreased in the SBSFL and FBSFL treatments compared to CBSFL treatment. A similar trend was observed for individual larval weight and larval length, which both decreased significantly in the order CBSFL, SBSFL and FBSFL. The larval height was significantly greater in CBSFL and SBSFL compared to FBSFL.

**Table 3:** Growth performance of *Hermetia illucens* larvae reared on food waste substrates supplemented with fish and soybean meal.

Experimental group	Total biomass (kg)	Final weight (g/larvae)	LL (mm/larvae)	LH (mm/larvae)
CBSFL	2.13 ± 0.12^a^	25.1 ± 0.3^a^	19.3 ± 0.3^a^	5.80 ± 0.06^a^
SBSFL	1.70 ± 0.05^b^	23.4 ± 0.2^b^	17.5 ± 0.2^b^	5.88 ± 0.02^a^
FBSFL	1.59 ± 0.05^b^	21.4 ± 0.3^c^	15.9 ± 0.3^c^	5.47 ± 0.03^b^

*Note*: The values represent the mean of triplicate groups (*n* = 3), presented as the mean ± SE. Values with different superscript letters in the same row are significantly different (*p*  < 0.05). The lack of superscript letters indicates no significant differences among the treatments. CBSFL, control (100% food waste); SBSFL, soybean meal‐supplemented substrate (70% food waste, 15% soybean meal and 15% water); FBSFL, fish meal‐supplemented substrate (70% food waste, 15% fish meal and 15% water).

Abbreviations: LH, larval height; LL, larval length.

### 3.2. BSFL Proximate Composition

Table 4 lists the nutrient analysis results of BSFL after 13 days of rearing on different substrates. The SBSFL group exhibited significantly lower levels of dry matter and crude ash compared to the CBSFL group (*p*  < 0.05). The contents of crude fat, calories and crude fibre were also significantly elevated in the CBSFL group relative to those in the SBSFL and FBSFL groups. Conversely, crude protein was significantly higher in the SBSFL and FBSFL groups than in the CBSFL group.

**Table 4:** Nutrient composition (%) of *Hermetia illucens* larvae reared on food waste substrates supplemented with fish and soybean meal.

Experimental group	Dry matter	Crude protein	Crude lipid	Ash	Calorie	Crude fibre
CBSFL	98.2 ± 0.6^a^	40.3 ± 0.4^b^	34.8 ± 0.0^a^	10.9 ± 0.2^a^	5914 ± 37^a^	7.4 ± 0.3^a^
SBSFL	95.4 ± 0.2^b^	48.0 ± 0.2^a^	29.2 ± 0.2^b^	8.6 ± 0.1^b^	5713 ± 23^b^	6.3 ± 0.1^b^
FBSFL	96.1 ± 0.2^ab^	47.8 ± 0.3^a^	28.4 ± 0.4^b^	10.1 ± 0.0^ab^	5655 ± 41^b^	6.0 ± 0.0^b^

*Note*: The values represent the mean of triplicate groups (*n* = 3), presented as the mean ± SE. Values with different superscript letters in the same row are significantly different (*p*  < 0.05). The lack of superscript letters indicates no significant differences among the treatments. CBSFL, control (100% food waste); SBSFL, soybean meal‐supplemented substrate (70% food waste, 15% soybean meal and 15% water); FBSFL, fish meal‐supplemented substrate (70% food waste, 15% fish meal and 15% water).

### 3.3. Bound Amino Acid Composition

Table 5 summarises the amino acid composition of the larvae. Among the essential amino acids (EAAs), lysine and phenylalanine contents were significantly higher in the SBSFL group than in the CBSFL group (*p*  < 0.05). The histidine content was also significantly higher in the SBSFL group than in both the FBSFL and CBSFL groups (*p*  < 0.05). No significant differences were observed for the other EAAs among the different substrates (*p*  > 0.05).

**Table 5:** Amino acid composition (% of the protein) of *Hermetia illucens* larvae reared on food waste substrates supplemented with fish and soybean meal.

Components	Experimental group		
	CBSFL	SBSFL	FBSFL
Essential amino acids (% of the protein)			
Histidine	3.37 ± 0.05^b^	3.73 ± 0.02^a^	3.47 ± 0.01^b^
Lysine	6.02 ± 0.16^b^	6.62 ± 0.04^a^	6.44 ± 0.07^ab^
Phenylalanine	4.10 ± 0.05^b^	4.49 ± 0.11^a^	4.42 ± 0.07^ab^
Methionine	1.22 ± 0.00	1.28 ± 0.06	1.33 ± 0.01
Isoleucine	4.59 ± 0.08	4.42 ± 0.01	4.51 ± 0.02
Leucine	7.30 ± 0.09	6.99 ± 0.01	7.13 ± 0.02
Threonine	3.95 ± 0.06	4.03 ± 0.01	4.01 ± 0.02
Valine	6.24 ± 0.03	6.26 ± 0.02	6.16 ± 0.12
Arginine	5.09 ± 0.09	5.14 ± 0.05	5.19 ± 0.01
Non‐essential amino acids (% of the protein)			
Cystine	0.66 ± 0.07	0.58 ± 0.05	0.61 ± 0.09
Glycine	5.50 ± 0.11	5.39 ± 0.02	5.54 ± 0.01
Alanine	6.56 ± 0.19^a^	5.88 ± 0.03^b^	6.05 ± 0.09^ab^
Aspartic acid	9.92 ± 0.14^b^	10.70 ± 0.06^a^	10.46 ± 0.03^a^
Serine	4.25 ± 0.07	4.03 ± 0.03	4.04 ± 0.04
Glutamic	11.67 ± 0.12^ab^	11.30 ± 0.11^b^	11.96 ± 0.15^a^
Proline	5.41 ± 0.27^a^	4.85 ± 0.23^ab^	4.42 ± 0.09^b^
Tyrosine	6.46 ± 0.09^b^	7.28 ± 0.14^a^	6.61 ± 0.02^b^
Ammonia	7.68 ± 0.14^a^	7.04 ± 0.07^b^	7.63 ± 0.13^a^

*Note*: The values represent the mean of triplicate groups (*n* = 3), presented as the mean ± SE. Values with different superscript letters in the same row are significantly different (*p*  < 0.05). The lack of superscript letters indicates no significant differences among the treatments. CBSFL, control (100% food waste); SBSFL, soybean meal‐supplemented substrate (70% food waste, 15% soybean meal and 15% water); FBSFL, fish meal‐supplemented substrate (70% food waste, 15% fish meal and 15% water).

For the non‐EAAs, the levels of aspartic acid and tyrosine were significantly higher in the SBSFL group than in the CBSFL group. In contrast, the proline content was significantly lower in the FBSFL group compared to the CBSFL group, whereas no significant difference was found between the SBSFL and CBSFL groups. The ammonia content was significantly higher in the CBSFL and FBSFL groups than in the SBSFL group. The glutamic acid content was significantly higher in the FBSFL than in the SBSFL group.

### 3.4. BSFL Fatty Acid Composition

The fatty acid composition of the BSFL was significantly affected by the dietary substrate (Table 6). The total saturated fatty acid (SFA) content was significantly higher in the CBSFL and SBSFL groups than in the FBSFL group. Specifically, the content of lauric acid (C12:0) was notably higher in the CBSFL and SBSFL groups, whereas the palmitic acid (C16:0) content was highest in the FBSFL group. In contrast, the total monounsaturated fatty acid (MUFA) content was significantly higher in the FBSFL group than in the other two groups. This was primarily driven by the significantly higher contents of oleic acid (C18:1n‐9), stearic acid (C18:0) and palmitoleic acid (C16:1) in the FBSFL group. No significant differences were observed in the total polyunsaturated fatty acid (PUFA) content among the experimental groups. However, among the individual PUFAs, the content of linoleic acid (C18:2n‐6), a major omega‐6 fatty acid, was highest in the SBSFL group, whereas the content of eicosapentaenoic acid (EPA; C20:5n‐3), an omega‐3 fatty acid, was highest in the FBSFL group.

**Table 6:** Major fatty acid composition (% of the total fatty acids) of *Hermetia illucens* larvae reared on food waste substrates supplemented with fish and soybean meal.

Components	Experimental group		
	CBSFL	SBSFL	FBSFL
C10:0	2.45 ± 0.10^a^	2.11 ± 0.06^b^	1.99 ± 0.05^b^
C12:0	47.2 ± 0.5^a^	44.7 ± 0.8^a^	35.9 ± 1.9^b^
C14:0	5.71 ± 0.25^b^	6.94 ± 0.11^a^	6.72 ± 0.36^ab^
C16:0	12.75 ± 0.37^b^	11.59 ± 0.36^b^	15.97 ± 0.32^a^
C16:1	2.28 ± 0.05^b^	1.84 ± 0.01^b^	3.31 ± 0.20^a^
C18:0	2.74 ± 0.11^b^	3.69 ± 0.13^ab^	4.31 ± 0.45^a^
C18:1n‐9	13.82 ± 0.27^b^	14.15 ± 0.48^b^	16.84 ± 0.29^a^
C18:2n‐6	11.05 ± 0.07^b^	12.79 ± 0.18^a^	9.64 ± 0.39^c^
C18:3n‐3	1.26 ± 0.14	1.51 ± 0.10	1.67 ± 0.18
C20:5n‐3	0.69 ± 0.03^b^	0.55 ± 0.05^b^	3.20 ± 0.12^a^
SFA	70.8 ± 0.2^a^	69.0 ± 0.5^a^	64.9 ± 0.9^b^
MUFA	18.8 ± 0.4^b^	19.7 ± 0.6^b^	24.5 ± 0.7^a^
PUFA	13.01 ± 0.06	14.85 ± 0.07	14.50 ± 0.68

*Note*: The values represent the mean of triplicate groups (*n* = 3), presented as the mean ± SE. Values with different superscript letters in the same row are significantly different (*p*  < 0.05). The lack of superscript letters indicates no significant differences among the treatments. CBSFL, control (100% food waste); SBSFL, soybean meal‐supplemented substrate (70% food waste, 15% soybean meal and 15% water); FBSFL, fish meal‐supplemented substrate (70% food waste, 15% fish meal and 15% water).

Abbreviations: MUFA, monounsaturated fatty acids; PUFA, polyunsaturated fatty acids; SFA, saturated fatty acids.

### 3.5. Olive Flounder Growth Performance

No significant differences were observed in survival rate, feed utilisation (feed conversion ratio and daily feed intake), or somatic indices (condition factor, hepatosomatic index and viscerosomatic index) among the dietary groups (*p*  > 0.05) (Table 7). In contrast, growth performance (weight gain and specific growth rate) of fish fed the CBSFL14 diet was significantly lower than that of all other groups, including the CON, 7% inclusion groups (CBSFL7 and SBSFL7) and SBSFL14 group (*p*  < 0.05).

**Table 7:** Effects of black soldier fly larvae (BSFL) meal dietary inclusion from different rearing substrates on the growth, feed utilisation and morphological indices of olive flounder (*Paralichthys olivaceus*).

Parameters	Experimental group				
	CON	CBSFL7	CBSFL14	SBSFL7	SBSFL14
SUR (%)	93.3 ± 2.7	97.3 ± 1.3	97.3 ± 1.3	96.0 ± 4.0	94.7 ± 3.5
WG (%)	367.7 ± 12.3^a^	385.1 ± 4.7^a^	326.1 ± 11.9^b^	376.5 ± 7.1^a^	345.5 ± 4.2^ab^
SGR (%/day)	2.75 ± 0.05^a^	2.82 ± 0.02^a^	2.59 ± 0.05^b^	2.79 ± 0.03^a^	2.67 ± 0.02^ab^
FCR	1.02 ± 0.03	1.07 ± 0.03	1.02 ± 0.01	1.09 ± 0.04	1.03 ± 0.04
DFI (%)	1.37 ± 0.04	1.43 ± 0.05	1.26 ± 0.04	1.35 ± 0.00	1.30 ± 0.05
CF	0.92 ± 0.01	0.92 ± 0.02	0.91 ± 0.01	0.93 ± 0.01	0.90 ± 0.01
HSI	1.02 ± 0.01	1.00 ± 0.02	1.03 ± 0.04	1.04 ± 0.03	1.03 ± 0.09
VSI	2.86 ± 0.05	2.99 ± 0.13	3.17 ± 0.06	3.09 ± 0.03	3.18 ± 0.03

*Note*: The values represent the mean of triplicate groups (*n* = 3), presented as the mean ± SE. Values with different superscript letters in the same row are significantly different (*p*  < 0.05). The lack of superscript letters indicates no significant differences among the treatments. FM, 60% fish meal; CBSFL7, 53% fish meal + 7% CBSFL; CBSFL14, 46% fish meal + 14% CBSFL; SBSFL7, 53% fish meal + 7% SBSFL; SBSFL14, 46% fish meal + 14% SBSFL.

Abbreviations: CF, condition factor; DFI, daily feed intake; FCR, feed conversion ratio; HSI, hepatosomatic index; SGR, specific growth rate; SUR, survival rate; VSI, viscerosomatic index; WG, weight gain.

### 3.6. Dorsal Muscle Proximate and Amino Acid Composition

Table 8 lists the proximate, EAA and fatty acid compositions of the flounder dorsal muscle. Concerning the proximate composition, no significant differences were found among the dietary treatments in the moisture, crude protein, and ash contents. However, the crude lipid content was significantly higher in CON and CBSFL14 than in the other treatments. For the EAA composition, the contents of histidine, lysine, methionine, threonine and arginine displayed no significant differences among all dietary treatments. In contrast, significant differences were observed for several other amino acids. The phenylalanine content was significantly higher in CBSFL14, SBSFL7 and SBSFL14 than in CON, although it was not significantly different from that in CBSFL7. The isoleucine content was significantly lower in the SBSFL7 compared to the CON, although it did not exhibit significant differences among the other treatments. The leucine content was significantly lower in CBSFL14 and SBSFL14 compared to CON, CBSFL7 and SBSFL7. The valine content was significantly lower in CBSFL14 and SBSFL7 compared to CON, while CBSFL7 and SBSFL14 showed no significant difference from CON. The dietary inclusion of BSFL significantly altered the major fatty acid composition of the flounder dorsal muscle. Fish fed the BSFL diets exhibited significantly higher proportions of SFAs and MUFAs compared to CON (*p*  < 0.05). Specifically, all BSFL treatments showed significantly higher levels of C12:0 and C14:0 than the control treatment. Conversely, fish fed the high‐inclusion diets (CBSFL14 and SBSFL14) presented significantly lower contents of essential PUFAs, such as C18:2n‐6 and C22:6n‐3, compared to CON.

**Table 8:** Effects of dietary inclusion of black soldier fly larvae (BSFL) meal from different rearing substrates on proximate composition, essential amino acid composition and fatty acid composition of dorsal muscle of olive flounder (*Paralichthys olivaceus*).

Components	Experimental groups				
	CON	CBSFL7	CBSFL14	SBSFL7	SBSFL14
Proximate composition (%)					
Moisture	76.2 ± 0.1	75.9 ± 0.3	76.0 ± 0.1	75.9 ± 0.1	75.7 ± 0.1
Crude protein	21.8 ± 0.2	22.5 ± 0.3	22.2 ± 0.2	22.2 ± 0.1	22.6 ± 0.1
Crude lipids	0.47 ± 0.02^a^	0.30 ± 0.02^b^	0.46 ± 0.04^a^	0.25 ± 0.03^b^	0.21 ± 0.03^b^
Ash	1.44 ± 0.00^ns^	1.46 ± 0.04	1.41 ± 0.01	1.48 ± 0.00	1.45 ± 0.00
Essential amino acid composition (% of the protein)					
Histidine	2.19 ± 0.02	2.21 ± 0.02	2.18 ± 0.02	2.20 ± 0.03	2.18 ± 0.02
Lysine	9.92 ± 0.13	9.89 ± 0.10	9.88 ± 0.14	10.36 ± 0.29	9.86 ± 0.25
Phenylalanine	3.86 ± 0.02^b^	4.01 ± 0.05^ab^	4.05 ± 0.03^a^	4.06 ± 0.04^a^	4.07 ± 0.03^a^
Methionine	2.71 ± 0.03	2.71 ± 0.01	2.64 ± 0.02	2.67 ± 0.04	2.56 ± 0.07
Isoleucine	4.88 ± 0.04^a^	4.65 ± 0.10^ab^	4.70 ± 0.05^ab^	4.55 ± 0.03^b^	4.76 ± 0.04^ab^
Leucine	8.21 ± 0.04^a^	8.26 ± 0.05^a^	8.03 ± 0.03^b^	8.23 ± 0.03^a^	8.04 ± 0.02^b^
Threonine	4.01 ± 0.14	4.38 ± 0.09	4.50 ± 0.02	4.55 ± 0.03	4.47 ± 0.03
Valine	5.39 ± 0.04^a^	5.18 ± 0.05^ab^	5.15 ± 0.08^b^	5.10 ± 0.02^b^	5.20 ± 0.03^ab^
Arginine	5.50 ± 0.15	5.49 ± 0.28	5.37 ± 0.19	5.10 ± 0.32	5.21 ± 0.31
Major fatty acid composition (% of the total fatty acids)					
C12:0	0.12 ± 0.02^c^	2.02 ± 0.12^b^	4.28 ± 0.18^a^	2.13 ± 0.07^b^	4.47 ± 0.20^a^
C14:0	2.09 ± 0.02^c^	2.93 ± 0.02^b^	3.52 ± 0.05^a^	2.93 ± 0.03^b^	3.58 ± 0.08^a^
C16:0	22.6 ± 0.1^a^	22.0 ± 0.5^a^	20.7 ± 0.2^b^	19.9 ± 0.1^b^	19.8 ± 0.1^b^
C16:1	2.30 ± 0.1^b^	3.00 ± 0.1^a^	2.95 ± 0.0^a^	2.84 ± 0.0^a^	2.90 ± 0.0^a^
C18:0	6.71 ± 0.11^a^	6.56 ± 0.05^a^	6.46 ± 0.04^ab^	6.02 ± 0.04^c^	6.21 ± 0.05^bc^
C18:1n‐9	15.5 ± 0.0^b^	16.2 ± 0.1^ab^	16.2 ± 0.1^a^	15.7 ± 0.1^ab^	15.6 ± 0.1^ab^
C18:2n‐6	18.2 ± 0.0^a^	16.9 ± 0.1^c^	13.8 ± 0.1^d^	17.6 ± 0.1^b^	13.8 ± 0.0^d^
C20:5n‐3	7.40 ± 0.03^ab^	7.46 ± 0.03^ab^	7.50 ± 0.05^ab^	7.11 ± 0.15^b^	7.63 ± 0.10^a^
C22:6n‐3	14.1 ± 0.1^a^	13.4 ± 0.3^ab^	12.3 ± 0.2^b^	12.2 ± 0.2^b^	12.1 ± 0.3^b^
SFA	34.4 ± 0.2^c^	36.7 ± 0.6^b^	39.2 ± 0.1^a^	34.5 ± 0.5^c^	38.4 ± 0.2^ab^
MUFA	17.8 ± 0.1^b^	19.2 ± 0.2^a^	19.2 ± 0.1^a^	18.7 ± 0.1^ab^	19.2 ± 0.3^a^
PUFA	43.2 ± 0.2^a^	39.8 ± 0.7^b^	36.4 ± 0.2^c^	41.4 ± 0.6^ab^	37.1 ± 0.3^c^

*Note*: The values represent the mean of triplicate groups (*n* = 3), presented as the mean ± SE. Values with different superscript letters in the same row are significantly different (*p*  < 0.05). The lack of superscript letters indicates no significant differences among the treatments. FM, 60% fish meal; CBSFL7, 53% fish meal + 7% CBSFL; CBSFL14, 46% fish meal + 14% CBSFL; SBSFL7, 53 % fish meal + 7% SBSFL; SBSFL14, 46% fish meal + 14% SBSFL.

Abbreviations: MUFA, monounsaturated fatty acids; PUFA, polyunsaturated fatty acids; SFA, saturated fatty acids.

## 4. Discussion

### 4.1. BSFL Growth Performance

Contrary to the general expectation that supplementing with high‐protein ingredients such as SBM or FM would enhance growth, larvae reared solely on FW exhibited superior growth performance. Moisture content is unlikely to explain the observed differences in larval growth. Substrate moisture content in the range of 50%–80% positively affects larval development and final weight [25–31]. Because the substrates used in this study had moisture levels of 74% and 85%, both within or near this optimal range, moisture was unlikely to be a primary inhibitory factor. Therefore, the results reveal that specific properties of the supplemented ingredients may have had an unexpectedly negative impact on larval growth. Several factors could explain the relatively poor performance of the supplemented groups.

First, for the CBSFL group, the growth inhibition may be related to the high lipid content of the substrate, consistent with previous reports describing that excessive dietary fat could delay larval development [32, 33]. The addition of 15% FM, which is itself lipid‐rich, likely excessively increased the total lipid level of the substrate, thereby impairing growth. Second, in the SBM‐supplemented group, anti‐nutritional factors, known to be present in SBM (such as trypsin inhibitor, haemagglutinin and raffinose) may have inhibited feed intake and digestion [34, 35]. Whether BSFL could secrete specific digestive enzymes to effectively degrade these anti‐nutritional factors remains unclear, a point that requires further investigation. The superior BSFL growth on the FW substrate is likely linked to the evolutionary adaptation of these animals. In the wild, BSFL have evolved to thrive on heterogeneous decaying matter, making the complex FW mixture an ideal substrate that is physiologically suited for them [33].

### 4.2. BSFL Nutrient Profile

The general BSFL composition revealed distinct differences according to the nutritional composition of the rearing substrate. Larvae reared on substrates containing high‐protein ingredients, namely SBM (crude protein, 5%) and FM (crude protein, 68%), exhibited higher crude protein content than those reared on a sole FW (crude protein, 4.5%) substrate. This result reflects the dietary protein level on the body composition of the larvae. However, this result contradicts previous studies that reported that larvae fed low‐protein diets accumulated a higher protein content [36–38]. This discrepancy could stem from different experimental conditions, such as the specific type of substrate, rearing density, and experimental duration. In contrast, the CBSFL group had the highest crude lipid content among all experimental groups. This is likely attributed to the biological ability of BSFL to efficiently convert carbohydrates and fibre into body lipids [33, 39, 40], a feature that could be correlated with the fibre‐rich FW substrate. Furthermore, considering the tendency for larger individuals to accumulate more fat [33, 41, 42], the high lipid content in the CBSFL group indicates a possible influence from differences in individual body size resulting from rapid growth.

BSFL fatty acid composition is only partially influenced by the dietary substrate of the animals. This is because the larvae can biosynthesize certain SFAs, such as lauric acid (C12:0), directly from carbohydrates, even when absent from their diet [33, 43]. In the case of PUFAs; however, our study revealed that larvae are highly dependent on their diet. Specifically, when reared on a substrate containing FM, the content of the omega‐3 fatty acid EPA (C20:5n‐3) increased markedly. Considering the 48 h starvation period before analysis, this result is a significant finding, as it indicates that EPA was successfully incorporated into the larval tissues themselves, instead of simply being present in their gut contents. The content of n‐3 fatty acids in larvae is crucial for the growth and survival of farmed fish and, consequently, for the health of human consumers [44, 45]. For example, St‐Hilaire et al. [19] reported that rainbow trout fed a BSFL‐containing diet showed decreased levels of major n‐3 fatty acids (C20:5n‐3 and C22:6n‐3) in their tissues. This highlights that low n‐3 fatty acid content in standard BSFL‐based feed can be a significant obstacle to the sustainability of the aquaculture industry [46]. Utilising substrates derived from marine products, which are rich in n‐3 fatty acids, could be the most direct solution. However, this approach presents a significant trade‐off, as it could hinder larval growth, posing challenges for commercial application [47]. To overcome this issue, some studies have actively explored enriching n‐3 fatty acids through alternative substrates, such as microalgae waste [48] or silkworm pupae [49]. In conclusion, to maximise the value of BSFL as a future aquaculture feed, the key challenge is to simultaneously achieve two conflicting goals of rapid growth and high nutritional value. Therefore, subsequent research is urgently required to identify cost‐effective alternative substrates and to develop processes that can optimise both larval growth and fatty acid accumulation.

Consistent with Oonincx and Finke [50], the amino acid profile of the larvae in our study was strictly regulated and largely unaffected by the substrate composition. However, certain amino acids, including histidine, lysine and proline, showed active metabolic regulation by the larvae that cannot be explained by the substrate content alone. Notably, in this study, larvae reared on a proline‐free FBSFL substrate exhibited the highest internal proline concentrations. Similarly, lysine reached its highest levels in larvae from the SBSFL substrate, which had the lowest lysine content. This paradoxical phenomenon is not unique to our study, as Eggink et al. [51] also reported that larvae reared on a low‐proline mitigation mussel substrate had higher internal proline concentrations than those reared on a high‐proline shrimp waste substrate. However, the balance of specific amino acids could still critically influence larval growth. Lemme and Klüber [52] reported that although a reduction in other amino acids did not affect growth, methionine supplementation significantly improved the larval growth rate. This indicates that despite the larva’s robust regulatory capabilities, methionine acts as a limiting amino acid for maximal growth. In contrast, Koethe et al. [53] reported that excessively high dietary lysine significantly reduced larval survival, revealing that an oversupply of certain amino acids can exert toxic effects. These findings, which are unlikely to result solely from the effects of undigested gut content, reveal the existence of sophisticated metabolic pathways and inherent amino acid requirements in the larvae, independent of the dietary supply. Therefore, future research aimed at elucidating these biosynthetic and regulatory mechanisms at a molecular level is essential for maximising the feed efficiency of the BSF.

### 4.3. Olive Flounder Growth Performance and Feed Utilisation

In the present study, replacing up to 7% of FM with BSFL meal did not negatively affect the growth performance of olive flounder, regardless of the rearing substrate (CBSFL and SBSFL) used for the BSFL. This 7% replacement level is a conservative yet significant finding when compared with results reported for other carnivorous fish. For instance, replacing 100 g/kg of FM with 150 g/kg of BSFL meal had no impact on the growth or sensory quality of Atlantic salmon [54]. Similarly, Magalhães et al. [55] successfully replaced up to 22.5% of total dietary protein in European seabass, whereas Zhou et al. [56] included up to 140 g/kg (14%) BSFL meal in Jian carp without adverse effects on growth. However, the optimal inclusion level of BSFL meal appears to be highly dependent on the feeding habits of the species, which supports the lower replacement rate observed in carnivorous fish such as olive flounder compared with omnivorous species. For example, Limbu et al. [57] reported that replacing 81%–84% of FM with BSFL meal (corresponding to a dietary inclusion of ~34%–35%) improved growth performance of Nile tilapia by enhancing nutrient utilisation, without affecting survival rates. This stark difference is believed to be closely related to the digestive physiology of the species, particularly the activity of the enzyme chitinase, which breaks down chitin from the insect exoskeleton. When chitinase activity is present in fish, ingested chitin can serve a key nutritional function, contributing to energy intake [58–60]. However, carnivorous species such as olive flounder appear to have low secretion or activity of this enzyme, making it difficult for them to efficiently digest large quantities of BSFL meal. Therefore, for flounder diets that still rely on a high FM content of 60%, a 7% replacement level is considered a realistic inclusion rate that allows for the utilisation of BSFL’s nutritional benefits without negatively impacting growth.

Another key finding of this study is that the different rearing substrates (CBSFL and SBSFL) had no differential impact on flounder growth. This distinguishes our work from many previous studies that focused solely on the efficacy of BSFL from a single substrate. This result indicates that BSFL possesses a remarkable ability to standardise its nutritional composition regardless of the substrate consumed. This characteristic holds significant industrial importance, as it opens the possibility of using a wide range of locally available and low‐cost organic resources for BSFL production without compromising its quality as a feed ingredient.

Medium‐chain fatty acids (MCFAs) such as lauric acid (C12:0), which are abundant in BSFL, could be rapidly absorbed by cells and utilised as an efficient energy source and have been reported to promote growth in some fish species [61, 62]. In the present study, however, an increase in dietary MCFA did not positively affect olive flounder growth or feed efficiency. This finding is consistent with the results of a study on grouper by Tseng and Lin [63], who reported that growth tended to decrease when fish oil and soybean oil were replaced with MCFA‐rich coconut oil. This difference can be attributed to the fact that carnivorous marine fish, such as olive flounder, have a very low or non‐existent ability to convert short‐chain fatty acids into HUFAs de novo [22, 64, 65]. Therefore, unlike for freshwater species, such as Nile tilapia, meeting the requirement for essential fatty acids, such as n‐3 HUFAs, in marine species such as olive flounder is a more critical factor for growth than simply supplying a rapidly absorbed energy source (MCFAs). In this study, the n‐3 HUFA content of the experimental diets was ~9.5%–12.2%, which exceeded the requirement for juvenile olive flounder (7.96%–9.42%) reported by Kim and Lee [22]. In conclusion, under conditions where essential fatty acids were sufficiently supplied, the additional supplementation of MCFAs did not act as a further growth‐promoting factor. Therefore, when replacing FM with insect protein in aquafeeds for marine fish, a formulation strategy that prioritise meeting the n‐3 HUFA requirement over the MCFA content is of paramount importance.

### 4.4. Olive Flounder Nutrient and Amino Acid Composition

Feeding flounder with diets containing BSF meal reared on different substrates did not result in significant differences in the moisture, crude protein and crude ash contents of the fillets compared with those in the control group. However, the crude lipid content was significantly lower in all experimental groups supplemented with BSF meal compared with that in the control group, except for the CBSFL14 group. This decrease in fillet lipid content can be attributed to the hypolipidemic effect of chitin derived from BSF. Chitin, a major component of the insect exoskeleton, suppresses body lipid accumulation by inhibiting the digestion and absorption of dietary lipids, interfering with intestinal fatty acid synthesis, and disrupting the enterohepatic circulation of bile acids [55, 66]. Indeed, previous studies on rainbow trout (*Oncorhynchus mykiss*) and tongue sole (*Cynoglossus semilaevis*) have reported results similar to our findings, showing a decrease in intramuscular fat when fed diets containing BSF or chitin‐rich insect meal [41, 67]. However, this hypolipidemic effect is not consistently observed across all fish species, and outcomes can vary with experimental conditions, as exemplified by the lack of a significant difference in our CBSFL14 group [68, 69]. These inconsistencies reveal that the impact of chitin on lipid metabolism may depend on several factors, including the digestive physiology of the fish species, the concentration and physical form of chitin in the diet, and interactions with other dietary ingredients. Therefore, further research is required to elucidate the precise mechanisms through which BSF‐derived chitin affects lipid accumulation in fish.

An increase in the dietary content of isoleucine, leucine, valine, and phenylalanine led to a corresponding accumulation of these amino acids in the dorsal muscle of olive flounder. This result indicates that these EAAs supplied through the diet are directly utilised for muscle protein synthesis. These findings agree with Alam et al. [70], who reported that the whole‐body amino acid composition of juvenile flounder is influenced by dietary composition. However, the effect of dietary amino acids on body composition is not universal and can vary depending on the species and developmental stage. For instance, no direct correlation between the dietary and muscle content of specific amino acids was observed in studies on whiteleg shrimp (*Litopenaeus vannamei*) [71], tilapia (*Oreochromis niloticus*) [72] and juvenile olive flounder [73]. As pointed out by Wang et al. [74], such discrepancies could be attributed to differences in amino acid utilisation mechanisms arising from variations in species, body size, or feed ingredients. The practical significance of this study lies in confirming the efficacy of diets supplemented with BSF meal. Even in the experimental group with the lowest measured amino acid content, the composition of the dorsal muscle was comparable to levels reported in previous studies on olive flounder [75, 76].

### 4.5. Olive Flounder Fatty Acid Composition

The fatty acid composition of the diet is directly reflected in that of the fish muscle [77–79]. Based on this premise, the present study analysed the specific effects of BSFL meal as an FM replacer on the fatty acid profile of olive flounder dorsal muscle. As the BSFL meal inclusion level increased, the contents of lauric acid (C12:0) and total SFAs in the dorsal muscle increased proportionally and significantly. This is a direct reflection of the characteristics of the raw material, as BSFL is inherently rich in C12:0. This phenomenon of C12:0 accumulation has been reported in previous studies of other species, such as Atlantic salmon and Jian carp [54, 57]. Although an increase in SFAs in the muscle could improve shelf life owing to higher oxidative stability, excessive accumulation could dilute the relative proportion of functional fatty acids, such as n‐3 HUFAs, thereby affecting the nutritional value of the final product. In contrast, the nutritionally important n‐3 HUFAs showed opposing trends. Although the docosahexaenoic acid (DHA; C22:6n‐3) content significantly decreased with increasing BSFL inclusion, the EPA (C20:5n‐3) content showed no significant differences among all experimental groups. This difference reflects the absolute contents of these fatty acids in the respective diets. Considering the low dietary DHA levels in the BSFL‐supplemented diets and the limited biosynthetic capacity of olive flounder, the decrease in muscle DHA was expected. Conversely, it appears that a sufficient level of EPA (over 5.9% of total fatty acids) was supplied even in the BSFL diets to maintain its content in the muscle. Therefore, when utilising BSFL meal as an FM replacer, our results reveal that correcting the dietary DHA level, instead of the EPA level, is the key factor in maintaining the nutritional quality of farmed olive flounder. This is because DHA is recognised as a crucial quality indicator, being a functional component essential for the neural system development and overall health of human consumers.

In conclusion, considering that BSFL are naturally deficient in essential n‐3 fatty acids, a strategy of compensating for this nutritional gap by incorporating DHA‐rich ingredients, such as algal oil, represents an effective approach for designing high‐quality BSFL‐based feeds [13, 80, 81]. However, further evaluation of the cost‐effectiveness and in‐feed stability of such ingredients is required before commercial application.

## 5. Conclusion

This study highlights the dual potential of BSFL both in valorising organic waste and serving as a sustainable aquafeed ingredient. Our results indicate that although supplementing FW substrate with soybean or FM tends to increase the specific fatty acid and protein content inherent to the supplements, the use of non‐supplemented organic waste alone was the most efficient strategy for overall productivity, underpinning the importance of substrate selection that balances productivity with nutritional enrichment. Furthermore, this study established the BSFL meal produced through this process as a viable protein source for olive flounder. Dietary inclusion of up to 7% BSFL meal did not adversely affect growth performance or feed utilisation efficiency, thus showing high potential to replace conventional FM. Collectively, the findings provide a robust framework for a circular bioeconomy that transforms low‐value organic waste into a high‐value aquaculture supply. Future research should focus on optimising the organic waste pre‐treatment technology to maximise productivity and tailor the BSFL nutritional profile for specific purposes through substrate manipulation. In addition, it is essential to develop processing technologies to enhance the nutrient bioavailability of the final larval meal and verify its applicability to other major aquaculture species. These efforts will contribute to developing scalable and sustainable solutions for the interconnected global challenges in waste management and protein security.

## Funding

This research was funded by a grant from the National Institute of Fisheries Science, Republic of Korea (Grant R2026038).

## Disclosure

The sponsor had no role in relation to the study design, collection, analysis or interpretation of data, writing of the report and decision to submit the article for publication.

## Ethics Statement

The animal study protocol was approved by the Institutional Animal Care and Use Committee of the National Institute of Fisheries Science (Protocol Number 2024‐NIFS‐IACUC‐22; approved on 29 March 2024).

## Conflicts of Interest

The authors declare no conflicts of interest.

## Data Availability

The raw data supporting the conclusions of this article will be made available by the authors upon request.
